# Prognostic significance of JAM 3 in gastric cancer: An observational study from TCGA and GEO

**DOI:** 10.1097/MD.0000000000033603

**Published:** 2023-04-28

**Authors:** Qinfu Zhao, Jiayu Lian, Kai Pang, Ping Wang, Ruiyin Ge, Yanliu Chu

**Affiliations:** a Department of Gastroenterology, Weihai Municipal Hospital, Cheeloo College of Medicine, Shandong University, Weihai, Shandong Province, China; b Digestive Endoscopy Room, Weihai Municipal Hospital, Cheeloo College of Medicine, Shandong University, Weihai, Shandong Province, China; c Operation Management Section, Weihai Municipal Hospital, Cheeloo College of Medicine, Shandong University, Weihai, Shandong Province, China.

**Keywords:** immune cells, JAM3, methylation, stomach cancer, survival

## Abstract

Junctional adhesion molecule 3 (JAM3) can be used as a prognostic marker in multiple cancer types. However, the potential prognostic role of JAM3 in gastric cancer (GC) remains unclear. The purpose of this research was to gauge JAM3 expression and methylation as potential biomarkers for GC patient survival. Through bioinformatics research, we analyzed JAM3 expression, methylation, prognosis, and immune cell infiltrations. JAM3 methylation acts as a negative regulator of JAM3, leading to reduced expression of JAM3 in GC tissues relative to normal tissues. Patients with GC who expressed little JAM3 have a better chance of living a long time free of the disease, according to the Cancer Genome Atlas (TCGA) database. Through univariate and multivariate Cox regression analysis, inadequate JAM3 expression was labeled as an isolated indicator for overall survival (OS). The GSE84437 dataset was also used to confirm JAM3 prognostic role in GC, with consistent findings. A meta-analysis also found that low levels of JAM3 expression were significantly associated with longer OS. Finally, there was a strong correlation between JAM3 expression and a subset of immune cells. According to the TCGA database, low JAM3 expression could predict favorable OS and progression-free-survival (PFS) in GC patients (*P* < .05). The univariate and multivariate Cox regression demonstrated that low JAM3 expression was independent biomarker for OS (*P* < .05). Moreover, GSE84437 dataset was utilized to verify the prognostic role of JAM3 in GC, and the similar results were reached (*P* < .05). A meta-analysis revealed that low JAM3 expression was closely relevant to better OS. Finally, JAM3 expression exhibited a close correlation with some immune cells (*P* < .05). JAM3 might be a viable predictive biomarker and likely plays a crucial part in immune cell infiltration in individuals with GC.

## 1. Introduction

While it only accounts for 6.8% of all new cancer cases, gastric cancer (GC) is the fourth most prevalent malignancy overall and the second leading cause of cancer-related deaths globally.^[1]^ Moreover, the prognosis for GC patients is usually dismal.^[2,3]^ Most patients are detected at a late stage because of the lack of early particular symptoms, worsening the prognosis. The clinical outcomes of individuals with advanced stomach cancer remain disheartening, despite advancements in therapy.^[4,5]^ Only 5% of individuals with GC are reported to have a 5-year or more survival rate following prognosis, while GC accounts for 10% of all malignancies.^[6]^ Consequently, it is crucial to discover diagnostic and prognostic biomarkers that are both specific and sensitive for the early detection of GC in patients.

The tight junction function of epithelial and endothelial cells can be directly influenced by junction adhesion molecules (JAM), which are members of the immunoglobulin subfamily.^[7,8]^ Selective expression of the 6 members of the JAM subfamily (JAM1, JAM2, JAM3, JAM4, ESAM, and CAR) occurs in a wide variety of human tissues.^[9,10]^ According to previous research, JAM3 regulates both adhesion and transmigration. During tumor progression, JAM3 has been shown to play a vital role in controlling neoplasm growth.^[11–13]^ In lung squamous cell carcinoma cell, overexpression of JAM3 has been demonstrated to restore tight junctions and an epithelial phenotype.^[14]^ Even while JAM3 has been found to have a function in many different types of cancer, most studies have only revealed that it does so as a downstream pathway of other genes. The ability to utilize this as a standalone prognostic indicator in GC patients is not yet established. Additionally, JAM3 precise mechanism in GC, particularly its function in the TME, remains unclear.

Herein, we first compared the expression level and methylation status of JAM3 in GC samples with those in healthy subjects using bioinformatics analysis. The correlation between JAM3 methylation and expression and longevity was then studied. We also used information from the Cancer Genome Atlas (TCGA) and the Gene Expression Omnibus (GEO) to conduct a comprehensive meta-analysis of JAM3 predictive relevance. Additionally, the possible relationship between JAM3 and immune infiltration levels in GC was evaluated using the Tumor Immune Estimation Resource (TIMER) database.

## 2. Materials & methods

### 2.1. Data source

From the TCGA database (https://portal.gdc.cancer.gov/) we obtained clinical and transcriptome expression data for 375 GC tumor tissues and 32 GC normal tissues. The TCGA database was also mined for a genome-wide DNA methylation array. It was decided to get the GSE84437 dataset from GEO Database. TCGA and GEO belong to public databases. The patients involved in the database have obtained ethical approval. Users can download relevant data for free for research and publish relevant articles. Our study is based on open source data, so there are no ethical issues and other conflicts of interest. Following this, we used the R software to examine the TCGA data in order to determine the level of JAM3 expression in GC. Additionally, R statistical software was used to look at how much JAM3 was methylated.

### 2.2. Survival analysis in GC

RNA-seq and survival data from the TCGA were utilized in a survival analysis performed with the “Survminer,” “survivor” programs. ROC curves were built using the pROC and tmieROC packages in STAD. The GC data allowed for both univariate and multivariate cox regression analysis by R package. Using the GEO datasets, we confirmed that there was a correlation between JAM3 expression and GC outcome. We used a meta-analysis to evaluate the overall prognostic relevance of JAM3 in TCGA and GSE84437 GC patients. The association between JAM3 expression and the prognosis of GC patients was evaluated using a combined hazard ratio (HR) and 95%CI. The Q test was used to analyze the heterogeneity between TCGA and GEO datasets (*I*^2^ statistics). A random-effects model would be used for combination (*I*^2^ 50%) if there was no clear heterogeneity. If not, a fixed-effects model would be used instead. Meta-analysis was built using the STATA 15.1 program.

### 2.3. Correlation between JAM3 expression and immune cell infiltration in GC

To determine immune scores using mRNA expression data, the Estimation of Stromal and Immune cells in Malignant Tumors using Expression data (ESTIMATE) program was used. All 3 scores were derived from a single-sample gene set enrichment study. Among these were the stromal score, which showed the existence of tumor matrix, the immunological score, which indicated immune cell infiltration into the tumor, and the estimation score, which suggested tumor purity. To automatically analyze and visualize the relationship between immune infiltration levels and a set of factors, check out TIMER (https://cistrome.shinyapps.io/timer/).^[14]^ The TIMER method was used to determine whether or not there was a connection between the level of JAM3 expression and the numbers of 6 different immune cell types (B cells, CD4 + T cells, CD8 + T cells, dendritic cells, neutrophils, macrophages, and so on) in GC. We also investigated JAM3 prognostic utility in GC patients with varying degrees of immune cell abundance. In addition, we used the correlation module to estimate the relationship between JAM3 and the markers for the following cell types present in GC: T cells, CD8 + T cells, B cells, CD8 + T cells, neutrophils, monocytes, tumor-associated macrophages, M1 cells, M2 cells, NK cells, dendritic cells, and Treg cells.

### 2.4. Pathway enrichment analysis

At first, patients with GC from TCGA were split into high JAM3 expression and low JAM3 expression groups based on their level of JAM3 expression. With a false detection rate of <0.05, we chose genes that were differentially expressed between the 2 groups. We considered significant the presence of |logFC| ≥ 1 in gene terms with a *P* value of <.05. A gene set with a *P* value <.05 was considered significantly enriched in Kyoto Encyclopedia of Genes and Genomes and Gene Ontology terms.

### 2.5. Statistical analysis

Based on the median JAM3 mRNA expression value from the TCGA database, 2 groups, 1 with low and 1 with high JAM3 expression, were created. The correlations between JAM3 expression or DNA methylation and a set of nominal variables were examined using the chi-square or Fisher exact tests. Student *t* test was used to compare the means of 2 groups’ normally distributed continues indices, while a nonparametric test was used to examine the skewness of the distribution of their skew continues indices. The relationship between JAM3 expression and DNA methylation status was examined using the Pearson correlation coefficient. To further investigate whether JAM3 expression was an independent predictive factor in patients with GC, we used univariate and multivariate Cox regression models. The predictive value of JAM3 expression and JAM3 DNA methylation was assessed using Kaplan–Meier curves. The ability of JAM3 to predict 1-, 3-, and 5-year overall survival (OS) was analyzed using time-dependent receiver operating characteristic curves. The cutoff for statistical significance was set at a 2-tailed *P* value of <.05. The R (version 4.1.1), GraphPad Prism (version 9.0), and STATA (version 15.1) programs were used to examine the data.

## 3. Results

### 3.1. The expression and prognostic value of JAM3 in GC

**Figure 1.**
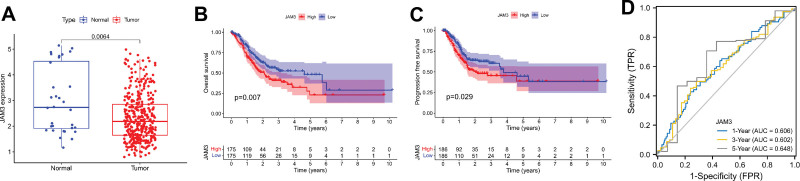
The expression and prognostic value of JAM3. (A) The expression of JAM3 in GC tissues and normal tissues; (B) Overall survival (OS) analysis between high JAM3 expression and low JAM3 expression in GC patients; (C) Progression-free survival (PFS) analysis between high JAM3 expression and low JAM3 expression in GC patients; (D) ROC curve of 1-, 3-, 5-yr survival of GC. GC = gastric cancer, JAM3 = junctional adhesion molecule 3.

**Figure 2.**
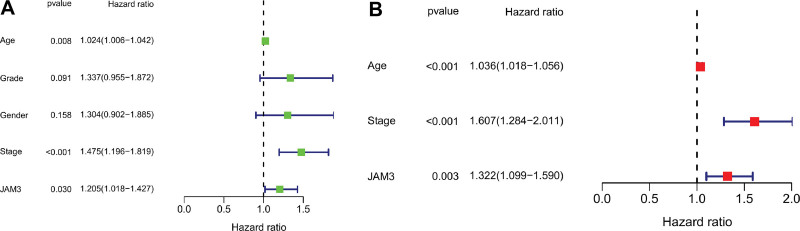
Univariate and multivariate Cox analysis. (A) Univariate Cox analysis and (B) multivariate cox analysis.

When we compared the mRNA expression levels of GC and normal tissues using data from the TCGA RNA-sequencing project (n = 375 GC tissues and 32 normal samples), we observed that JAM3 mRNA was significantly lower in GC tissues and significantly higher in normal tissues (*P* = .0064, Fig. 1A). Using the TCGA database, a survival study was performed to evaluate if there was a connection between JAM3 and prognostic significance in GC patients. Longer OS (*P* = .007) and progression-free-survival (PFS) (*P* = .029) were both negatively correlated with increased JAM3 expression in GC (Fig. 1B and C). Figure 1D demonstrated that JAM3 had an AUC of 0.606, 0.602, and 0.648 for predicting 1-year, 3-year, and 5-year OS rates of GC, respectively. Stage (HR: 1.475, 95% CI: 1.196–1.815, *P* < .001), and JAM3 expression (HR: 1.205, 95% CI: 1.018–1.427, *P* = .030) all stood as independent predictive indicators for GC in univariate analysis (Fig. 2A). In a multivariate analysis, we found that age (HR: 1.036, 95% CI: 1.018–1.056, *P* < .001), stage (HR: 1.607, 95% CI: 1.284–2.011, *P* < .001), and JAM3 expression (HR: 1.322, 95%CI: 1.099–1.590, *P* = .003) were all independent prognostic biomarkers (Fig. 2B).

### 3.2. The clinical and prognostic value of JAM3 methylation

**Figure 3.**
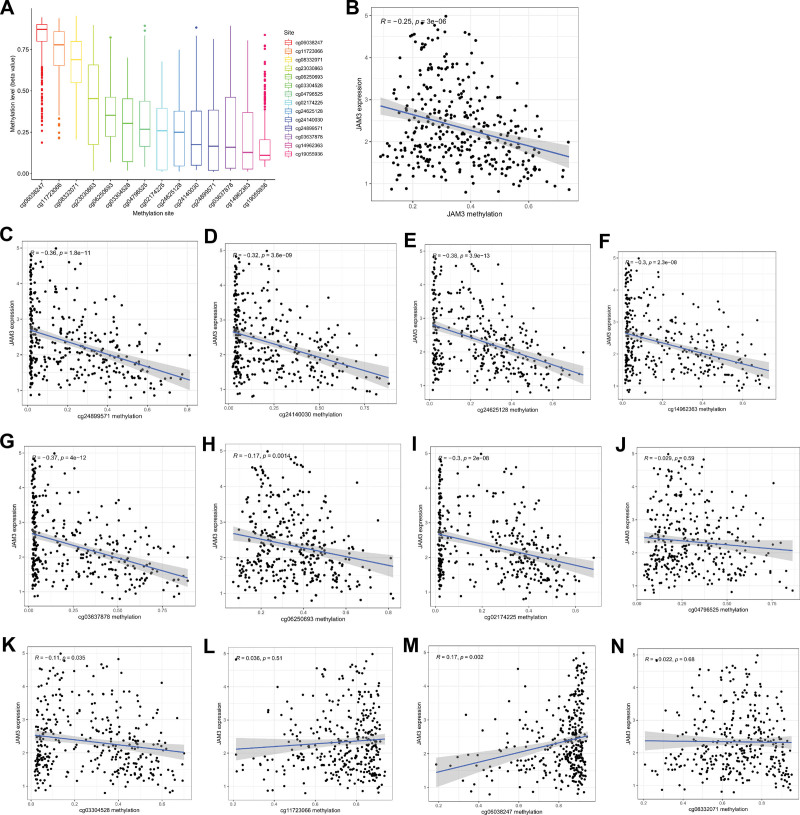
The methylation of JAM3 in GC. (A) The distribution of 14 JAM3 DNA promoter CpG sites. (B) The expression of JAM3 was negatively regulated by JAM3 DNA methylation. (C–N) The correlation of JAM3 expression with methylation of JAM3 DNA CpG sites. GC = gastric cancer, JAM3 = junctional adhesion molecule 3.

**Figure 4.**
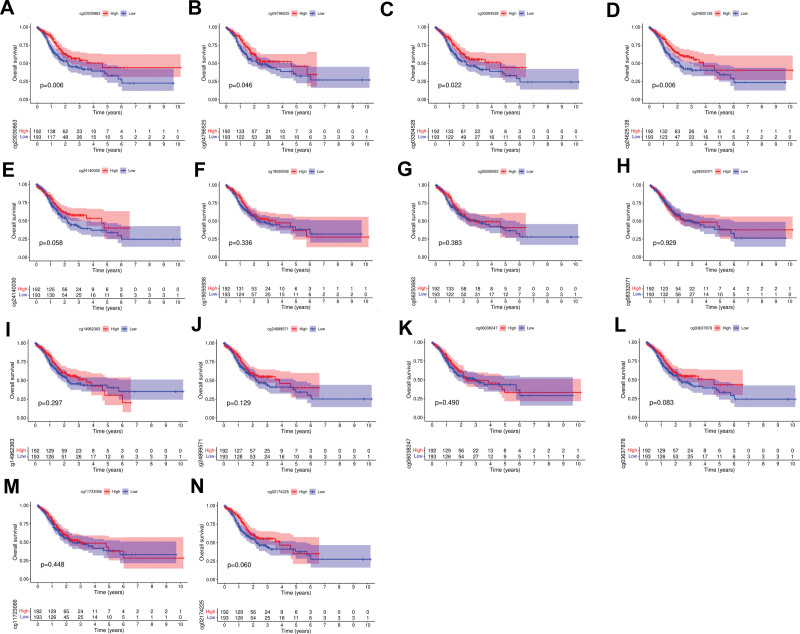
Overall survival curves of low and high JAM3 DNA promoter CpG sites in GC patients. GC = gastric cancer, JAM3 = junctional adhesion molecule 3.

**Figure 5.**
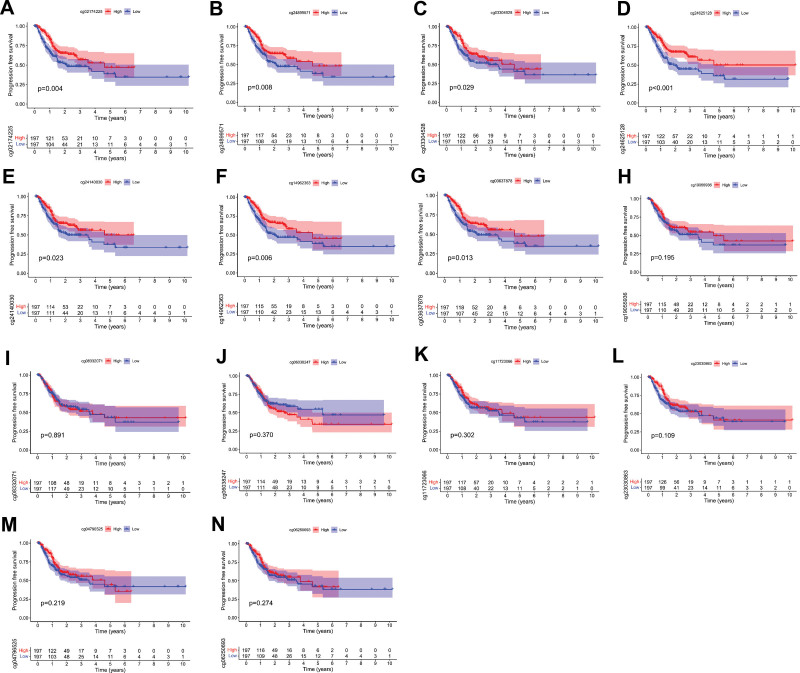
Progression-free survival curves of low and high JAM3 DNA promoter CpG sites in GC patients. GC = gastric cancer, JAM3 = junctional adhesion molecule 3.

In Figure 3A, we can see the dispersion of 14 different CpG sites in JAM3. The CpG sites in the JAM3 gene whose methylation was most negatively linked with mRNA expression were then isolated using Pearson correlation analysis (*R* = −0.25, *P* = 3e-06) (Fig. 3B). Methylation of all CpG sites except for 2 (cg11723066 and cg08332071) was significantly linked with JAM3 expression (Fig. 3C–N). To further understand the prognostic significance of these important JAM3 DNA CpG sites in GC patients, we first identified the relevant CpG sites. Kaplan–Meier plots showed that higher methylation at 3 CpG sites (cg23030863, cg04796525, cg03304528, and cg24625128) was associated with a better OS (Fig. 4A–D). However, no correlation was observed between methylation levels of ten CpG sites (cg24140030, cg19055936, cg05250693, cg08332071, cg14962363, cg24889571, cg0638247, cg0637878, cg11723066, and cg2174225) and OS among patients with GC (*P* > .05) (Fig. 4E–N). In addition, Figure 5A and G demonstrates that higher methylation levels at 7 CpG sites (cg02174225, cg24890571, cg03304528, cg24526128, cg24140030, cg14962363, and cg03637878) are associated with longer PFS in GC patients, while no correlation was found between methylation levels of another 7 CpG sites (cg19055936, cg08332071, cg06038247, cg11723066, cg23030863, cg04796525, and cg06250693) and PFS among patients with GC (*P* > .05) (Fig. 5H–N).

### 3.3. Validation of the prognostic value of JAM3 in GEO database

**Figure 6.**
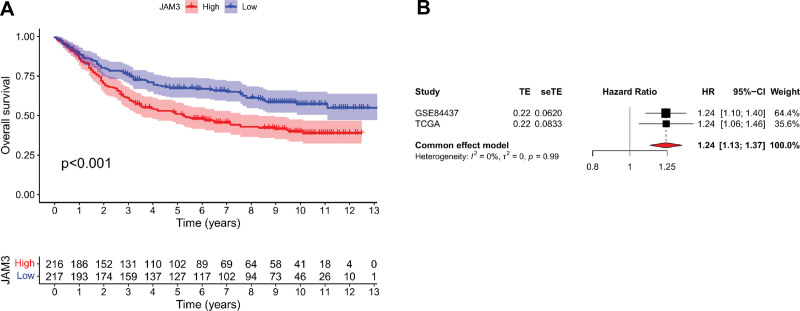
Survival validation of JAM3 expression in GC patients from GSE84437. (A) Survival analysis between high and low JAM3 expression in GC patients from GEO dataset. (B) Forest plot of low JAM3 expression with better OS in GC patients from TCGA and GEO datasets. GC = gastric cancer, JAM3 = junctional adhesion molecule 3, OS = overall survival.

For further confirmation of JAM3 prognostic importance, we evaluated microarray data from 433 GC patients from GSE84437. Using Kaplan–Meier analysis, we analyzed the correlation between JAM3 expression and OS in GC patients. In the GSE84437 dataset, low JAM3 was significantly associated with better OS than high JAM3 (*P* < .001) (Fig. 6A). We performed a meta-analysis using data from TCGA and GSE84437 since no prior research have demonstrated a connection between JAM3 expression and OS among patients with GC. There was no significant heterogeneity between the 2 datasets (*I*^2^ = 0%, *P* = .99), and the pooled HR for the link between high JAM3 expression and OS in 808 instances of GC was 1.24 (1.13–1.37) (Fig. 6B).

### 3.4. Relationships of JAM3 with tumor microenvironment

**Figure 7.**
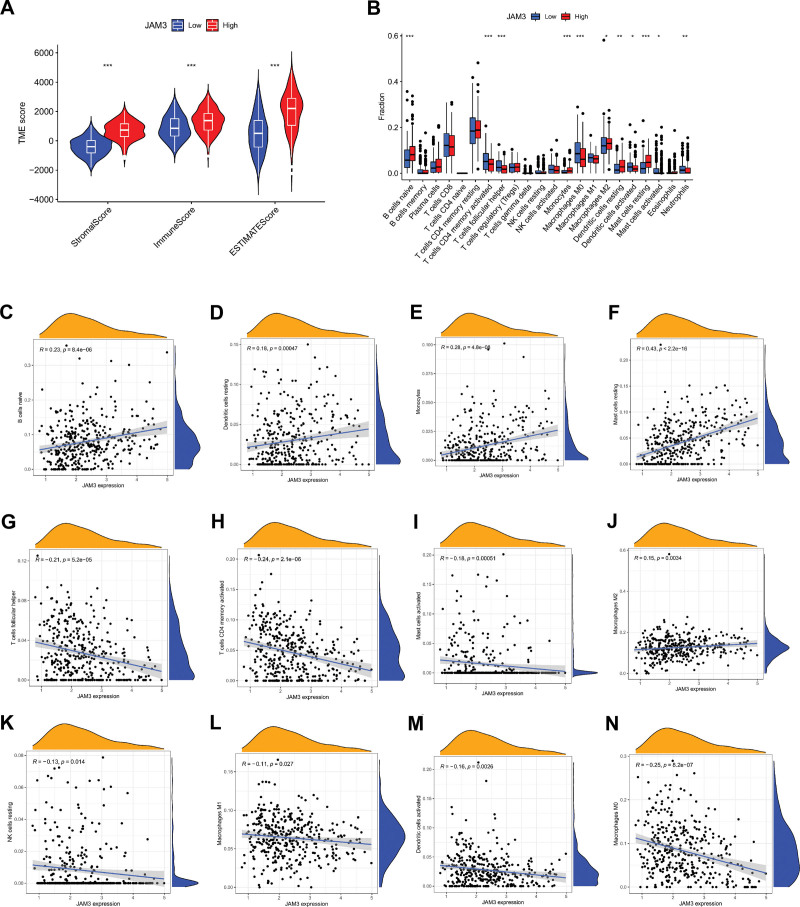
The correlation between JAM3 expression and tumor environment in GC. (A) The correlation between JAM3 expression and stromal score, immune score and ESTIMATE Score. (B) The relationship between JAM3 expression and multiple types of immune cell infiltration in GC. (C–N) The correlation between JAM3 expression and infiltration levels of immune cells in GC. ESTIMATE = Estimation of Stromal and Immune cells in Malignant Tumors using Expression data, GC = gastric cancer, JAM3 = junctional adhesion molecule 3.

375 GC patients had sufficient data for the use of immunocyte-related genes in the calculation of the stromal score, immunological score, and ESTIMATE Score. The results revealed that the stromal score, immunological score, and ESTIMATE Score were all better in patients with high JAM3 expression compared to those with low JAM3 expression (all *P* < .01) (Fig. 7A). Specifically, we looked into how JAM3 expression relates to immune cell infiltration in the GC tumor microenvironment. In Figure 7B, we can see that the expression of JAM3 was substantially linked to 11 subsets of the immune system cells. Five types of immune cells, including naive B cells, monocytes, M2 macrophages, resting dendritic cells, and resting mast cells, were less likely to infiltrate when JAM3 expression was higher, while 6 types of immune cells, including activated T cells, CD4 memory macrophages, M0 macrophages, activated dendritic cells, activated mast cells, and neutrophils, were more likely to do so (Fig. 7B). We dug deeper into the link between JAM3 and immune cell infiltration using the TIMER database. Specifically, JAM3 was shown to have a positive correlation with the infiltration of naïve B cells (*R* = 0.23, *P* = 8.4e-06), resting dendritic cells (*R* = 0.18, *P* = .00047), monocytes (*R* = 0.28, *P* = 4.8e-08), resting mast cells (*R* = 0.43, *P* = 2.2e-16), and Macrophages M2 (*R* = 0.15, *P* = .0034) (Fig. 7C–F and J) Seven different types of immune cells showed negative correlations with JAM3, including T follicular helper cells (r = −0.21, *P* = 5.2e-05), T memory activated cells (r = −0.24, *P* = 2.1e-06), activated Mast cells (r = −0.18, *P* = .00051), resting NK cells (r = −0.13, *P* = .014), activated Macrophages M1 (r = −0.11, *P* = .0; Fig. 7G–I and K–N).

### 3.5. JAM3 related signaling pathways in GC

**Figure 8.**
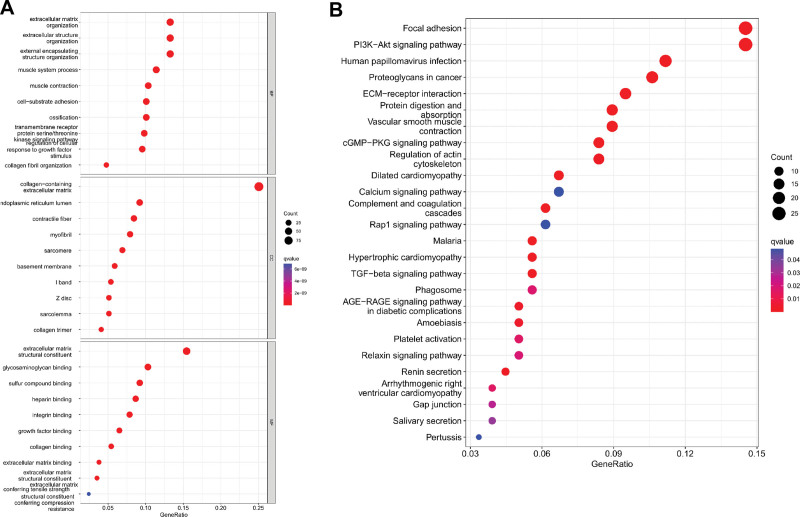
Pathway enrichment analysis. (A) GO analysis of differential genes between high JAM3 and low JAM3 expression group; (B) KEGG analysis of differential genes between high JAM3 and low JAM3 expression group. GO = gene ontology, JAM3 = junctional adhesion molecule 3, KEGG = Kyoto encyclopedia of genes and genomes.

The purpose of the functional enrichment study was to better understand the part that JAM3 plays in GC. Figure 8A displays the Gene Ontology together with the obtained results. JAM3 is involved in pathways of extracellular matrix organization, extracellular structure organization, cell substrate adhesion, collagen-containing extracellular matrix growth factor binding and so on (Fig. 8A). Focal adhesion, PI3K-Akt signaling pathway, Proteoglycans in cancer, ECM-receptor interaction, Protein digestion and absorption, cGMP-PKG signaling route, TGF-beta signaling pathway, and many more were found to be enriched for in the JAM3 Kyoto encyclopedia of genes and genomes pathway (Fig. 8B).

## 4. Discussion

The clinical and predictive value of JAM3 mRNA expression and JAM3 methylation in GC was investigated in our study. A substantial negative correlation was seen between JAM3 mRNA expression and JAM3 methylation, and low JAM3 mRNA expression in GC tissues. Cox models of survival analysis confirmed the importance of low JAM3 expression in predicting a good outcome for GC patients. Furthermore, we further confirmed the predictive function of JAM3 expression in GSE84437, and the results underscored the promising prognostic value of JAM3 expression in patients with GC. Low JAM3 expression was an independent predictive predictor of OS in our meta-analysis of 808 GC patients from TCGA and GSE84437. Our data also showed a strong correlation between JAM3 expression in GC and a number of immunological indicators, including the amount of immune infiltration. Our findings expand what is known about JAM3 possible function in the tumor immunology of GC and its prognostic importance.

Although several oncogenes have been found and confirmed as playing a role in the etiology of GC,^[15–19]^ much less is known regarding the clinical and prognostic significance of JAM genes. Tight junctions are a hotspot for JAMs because of their role in epithelial shape and migration. In order to create cell polarity in epithelial cells, tight junctions are essential, and previous research has shown that JAM3 is a key component of these junctions.^[20]^ There is a lot of nuance to JAM3 function in cancer. Tight connections were altered during tumor induction, allowing cells to bypass barriers created by intercellular junctions and adopt a more migratory phenotype.^[21]^ JAM3 has been shown to encourage the migration of renal cancer cell line^[22]^ and to inhibit apoptosis. Furthermore, a recent study indicated that JAM3 expression was present in numerous carcinoma cell lines, and this same study suggested that JAM3 was involved in tumor cell metastasis in lung cancer.^[23]^ The methylation or expression of JAM3 was also suggested as a potential new option for the noninvasive diagnosis of CRC^[24]^ in a research. However, JAM3 probable function in GC was unclear. Here, we used the TCGA and GEO databases to thoroughly investigate the correlation between JAM3 expression and survival. Patients with GC who expressed low levels of JAM3 had a dramatically better OS and PFS. Multivariate Cox regression confirmed these findings, demonstrating that low JAM3expression is a strong predictive predictor of OS in GC patients. Finally, we did a meta-analysis to pool data from 2 databases to determine JAM3 overall prognostic value; the results confirmed that decreased JAM3 expression was really linked to a better OS for GC patients. Taken as a whole, our data support the idea that JAM3 is a useful biomarker for predicting GC patient outcomes.

More and more corroboration alludes to a crucial role for aberrant DNA methylation in the initiation and development of GC.^[25–28]^ To begin our investigation, we used Pearson coefficients to see if the JAM3 methylation status affected JAM3 mRNA expression. In GC tissues, a strong inverse connection was seen between JAM3 methylation and JAM3 mRNA expression. The low levels of JAM3 expression in GC tissues may be explained by this inverse relationship. We next pinpointed the CpG sites in the JAM3 DNA promoter where methylation is highly linked with the expression level of mRNA. Intriguingly, with the exception of cg11723066 and cg08332071, nearly all of the CpG sites revealed significant relationships with JAM3 expression. Previous research has found a weak to moderate correlation between gene expression and DNA methylation, and it has only identified a small subset of genes that are highly controlled by DNA methylation.^[29,30]^ The predictive value of JAM3 DNA methylation was also explored. Among GC patients, we discovered that a hypermethylated JAM3 gene was associated with better OS and progression-free survival. Overall, JAM3 methylation status could be a strong indication of salutary OS and PFS, and JAM3 methylation adversely affected JAM3.

Previous research has shown that immune cells that infiltrate tumors are an important regulator of tumor development and progression in GC.^[31–33]^ We found that higher JAM3 expression was associated with increased infiltration of activated T cells, CD4 memory T cells, Macrophages M0, dendritic cells activated, mast cells activated, and neutrophiles and decreased infiltration of naive B cells, monocytes, Macrophages M2, resting dendritic cells, and resting mast cells. Additionally, JAM3 was found to have a favorable correlation with naïve B cells, resting dendritic cells, monocytes, resting Mast cells, and M2 macrophages. The levels of JAM3 were inversely linked with those of follicular helper T cells, activated memory T cells, active mast cells, resting NK cells, activated dendritic cells, and M1 and M0 macrophages. These findings showed that JAM3 regulates immune cells in GC, hence playing a role in the tumor immune microenvironment.

Despite the extensiveness of our investigation, there are still room for development. To begin, we emphasized that high and low JAM3 expression were merely relative terms, and that the criteria for defining either of these levels of expression had not yet been established. The criteria for high and low expression of JAM3 still need to be investigated. Second, because most of the patient data used in the article came from publicly available online sources, a lot of specifics were left out. Thirdly, GEO data did not back up the belief that JAM3 expression is linked to PFS, and only the TCGA database contained PFS data. That meant that a PFS meta-analysis was very unlikely to be conducted. So, more PFS data from larger samples was needed for further validation. However, the findings presented here are interesting and stand out in the search for GC prognostic biomarkers.

## 5. Conclusions

JAM3 might be a viable predictive biomarker and likely plays a crucial part in immune cell infiltration in individuals with GC, but need further research.

## Author contributions

**Conceptualization:** Qinfu Zhao, Yanliu Chu.

**Data curation:** Qinfu Zhao, Jiayu Lian, Ruiyin Ge.

**Formal analysis:** Qinfu Zhao, Kai Pang, Ping Wang.

**Methodology:** Qinfu Zhao, Kai Pang, Ping Wang.

**Writing – original draft:** Qinfu Zhao, Yanliu Chu.

**Writing – review & editing:** Qinfu Zhao, Yanliu Chu.
